# Tumour Cell Lines HT-29 and FaDu Produce Proinflammatory Cytokines and Activate Neutrophils In Vitro: Possible Applications for Neutrophil-Based Antitumour Treatment

**DOI:** 10.1155/2009/817498

**Published:** 2010-02-11

**Authors:** Antonio Brú, Juan-Carlos Souto, Sonia Alcolea, Rosa Antón, Angel Remacha, Mercedes Camacho, Marta Soler, Isabel Brú, Amelia Porres, Luis Vila

**Affiliations:** ^1^Applied Mathematics Department, Universidad Complutense de Madrid, 28040 Madrid, Spain; ^2^Haematology Department, Institute of Research of Hospital Santa Creu i Sant Pau, 08025 Barcelona, Spain; ^3^Laboratory of Angiology, Vascular Biology and Inflammation, Institute of Research of Hospital Santa Creu i Sant Pau, 08025 Barcelona, Spain; ^4^Haematology Department, Complejo Hospitalario Virgen de la Salud, 45004 Toledo, Spain; ^5^Centro de Salud La Estación, Talavera de la Reina, 45600 Toledo, Spain; ^6^Research Laboratory, Fundación Jiménez Díaz, 28040 Madrid, Spain

## Abstract

There is evidence that polymorphonuclear neutrophils (PMNs) can exert severe antineoplastic effects. Cross-talk between tumour cells and endothelial cells (ECs) is necessary for the accumulation of PMN around a tumour. This work reports the ability of two PMN-sensitive, human, permanent cell lines—colorectal adenocarcinoma (HT-29) and pharyngeal squamous-cell carcinoma (FaDu) cells—to act as inflammatory foci. PMNs were cytotoxic to both lines, the adhesion of the PMNs to the tumour cells being important in this effect. The tumour cells released appreciable amounts of IL-8 and GRO*α*, and induced the transmigration of PMN through human microvascular-EC monolayers. Conditioning media associated with both lines induced the adhesion of PMN and the surface expression of ICAM-1 in microvascular-EC. In addition, FaDu-conditioning-medium strongly induced the production of proinflammatory cytokines by microvascular-EC. These results support the idea that tumour cells might normally induce a potent acute inflammatory response, leading to their own destruction.

## 1. Introduction

Polymorphonuclear neutrophils (PMNs) are the most abundant circulating blood leukocytes. They play an important role in innate immunity and provide the first-line of defence against infection. Additionally, there is unequivocal evidence that PMN could play also an effective antitumour role. Edelson and Cohn reported in 1973 that lactoperoxidase exerts a cytotoxic activity against tumour cells [1]. Thereafter, Clark et al. [2] showed that peroxidase systems in inflammatory cells were cytotoxic for mouse lymphoma cells. During the next years, a number of reports appeared describing a cytotoxic effect of PMN and macrophages on cancer cells in vitro [3–9] in animal models and in humans [10–12]. It has been suggested that tumour progression is associated with neutropenia or a defective capacity of PMN to kill tumour cells [12–15]. It has been reported also that a mouse line, genetically selected for high acute inflammatory response exhibited an unusual resistance to tumourigenesis in comparison with the line selected for low acute inflammatory response. Interestingly, this mouse line produced large amounts of PMN in bone marrow, exhibited a high number of PMN in blood, and showed an increased resistance of the locally infiltrated PMN to spontaneously apoptose [16–19]. PMN were involved in the antitumour action of hyperthermia in an experimental mouse model [20]. PMN were involved also in the efficacy of the antitumour action following bacterial therapy with several classes of tumours [21–24]. In 2003, a mouse strain that was completely resistant to cancer was described [25]. Further, it had been demonstrated that this resistance was due to PMN, macrophages and NK cells [26]. The mechanism of this resistance is unknown, but it seems that the antitumour effect requires the contact between the immune cells and the malignant cells [27]. Similarly, Jaganjac et al. [28] showed that PMN were involved in the well-known spontaneous regression of W256 carcinoma cells grown in Sprage-Dawley rats. In addition, massive amounts of PMN were involved also in the rejection of tumour cells that were engineered to release the cytokines IL-2 and TNF*α* [29].

It is clear that PMN play an essential role in host defence against infection [30], but the mechanism by which PMN causes antitumour activity is not understood at all. It has been shown that the antitumour activity of inflammatory cells is mediated by a combination of oxidative and non-oxidative mechanisms. These complex mechanisms include intense production of reactive-oxygen species (ROS), hypoclorous acid, proteases, defensins, cytostatic factors, perforins, and membrane interactions between PMN and target cells [6–9, 31–36]. 

While studying tumour growth dynamics mathematically, using fractal geometry and scaling techniques, Brú et al. [37] found that growth of a solid tumour follows a universal growth pattern known as “linear molecular beam epitaxy.” This kind of growth dynamics is described by a differential equation, which contains a term that accounts for the diffusion of cells at the surface of the growing cell colony. The biological interpretation of this term led to the concept that, when a minimum of nutrients and oxygen are maintained, tumour cells prioritizes space and compete for it [38]. Based upon this concept, competition of inflammatory cells for the space around the solid tumour was proposed as mechanism that could contribute also to PMN antitumour activity. To test the antitumour effect of a massive infiltration of PMN, a sustained treatment with GM-CSF was applied in a mouse experimental model, and a strong neutrophilia was achieved around the induced tumours. Significantly, there was 16 times lower mortality in the treated mice than in the controls [39]. The same therapeutic strategy was applied in a patient with advanced hepatocarcinoma, who exhibited a complete remission after 4 months of G-CSF treatment [40]. There is indirect evidence by Su et al. [41] suggesting that the powerful antitumour effect of treatment with G-CSF is due to the up-regulation of the PMN production in bone marrow. These authors induced a sustained increase in the number of circulating PMN, by means of prolonged administration of G-CSF, and they found an unexpected survival rate in their double-blind, placebo-controlled randomized trial for squamous head and neck cancer. Patients in the G-CSF arm showed a mean leukocyte count of 24100/*μ*L during treatment period (around 50 days) and their 5-year disease-free survival was 84%. In comparison, the control group had a mean leukocyte count of 4100/*μ*L with a survival rate at 5 years of 47%. Other clinical trials using continuous GM-CSF administration in advanced prostate cancer [42] or sustained G-CSF in stage IV melanoma with brain metastases [43] reported a much better survival than when approved therapies were used.

Our hypothesis is that the efficiency of PMN against a tumour in vivo depends chiefly on the ability of the tumour to act as an inflammatory site to guarantee the recruitment of cells (especially PMN) to produce a potent acute inflammatory response. Recruitment of PMN from the blood by an inflammatory site is a multi-step process involving a series of coordinated interactions between PMN and endothelial cells (EC) [44]. Some pro-inflammatory mediators, such as cytokines, growth factors, lipid mediators, and so forth, secreted by the cells from the inflammatory site, activate EC which express adhesion molecules and elicit the production of downstream mediators from EC. In the case of tumours, the communication between tumour cells and EC should be the first step that initiates PMN accumulation at the site of tumour. By means of diapedesis, the PMN pass throughout the vessel wall and, to exert an antitumour effect they migrate towards the tumour cells and adhere to them. Finally, PMN are activated to secret cytokines that help orchestrate the antitumour immune response. Thus, our studies using two different tumour cell lines demonstrate that the tumours can be inflammatory sites that recruit PMN to which they are sensitive.

## 2. Material and Methods

### 2.1. Tumour Cell Lines

HT-29 and FaDu are permanent colorectal adenocarcinoma and pharinx scamous cell carcinoma cell lines respectively (obtained from American Type Culture Collection-ATCC HTB-38 and ATCC HTB-43) were grown in DMEM containing 10% foetal bovine serum (FBS) and supplemented with 2 mM L-glutamine, 1 mM sodium pyruvate, 100 U/mL penicillin and 100 *μ*g/mL streptomycin (Biological Industries, Kibutz Bet Haemek, Israel).

### 2.2. Isolation of PMN

PMN suspensions were obtained as previously described [45] from heparin anticoagulated peripheral venous blood from haematological patients suffering from iron-overload and who were undergoing periodic phlebotomies, following their signed consent. Cell viability, measured by Trypan blue dye exclusion, always exceeded 95%. Total and differential cell counts were carried out using Sysmex XE-2100 (Roche Spain, Barcelona, Spain) cell counter. In addition, the percentage of PMN were assessed using staining smears. The percentage was always higher than 95%.

### 2.3. Isolation and Culture of Human Microvascular Endothelial Cells (HMVEC)

Human dermal microvascular endothelial cells (HMVEC) were isolated from human foreskins by a modification of a previously described technique [46]. Briefly, foreskins were cut into 3 mm^2^ squares and placed in PBS containing 0.3% trypsin (Difco laboratories, Detroit, MI) and 1% EDTA (Sigma-Aldrich Química S.A. Madrid, Spain) at 37°C for 30 minutes. After washing the skin fragments with PBS several times, the cells were released by pressing into a Petri dish containing medium. The microvascular segments were passed through a 150 *μ*m nylon mesh and collected. HMVEC were cultured on 1% gelatine-coated flasks in MCDB 131 supplemented with 20% FBS, 20 mM L-glutamine, 100 U/mL penicillin, 100 *μ*g/mL streptomycin, 5 ng/mL bFGF, 20 ng/mL EGF (all from Biological Industries) and 10 U/mL heparin (Sigma). At first passage, HMVEC were purified with CD31-coated Dynabeads (Invitrogen Dynal AS, Oslo, Norway) following the manufacturer's instructions to obtain pure cell populations of HMVEC. The microvascular endothelial cells were identified by immunofluorescence staining for vWF and CD31. The experiments were conducted with cells that have been cultured by 3–5 passages. Prior to the experiments, cells were maintained for 48 hours in medium containing 1% FBS without heparin and without growth factors.

### 2.4. PMN Adhesion and Transendothelial Migration Assays

PMNs were suspended in PBS at cell density of 1 × 10^6^ PMN/mL. 1,1′-dioctadecyl-3,3,3′,3′-tetramethyl-indocarbocyanine perchlorate (Dil) in ethanol was added to yield 1 *μ*g/mL final concentration and incubated at 37°C for 1 hour. Afterwards, PMN were washed twice with PBS and suspended in DMEM containing 1% FBS at the adequate cell density. The labelled PMN suspension was warmed at 37°C for 5  minutes before the assays.

The adhesion assay was performed on tumour cells and HMVEC cultured in 12-well dishes at confluence. Cultured cells were washed twice before the addition of 0.5 mL of DMEM with 1% FBS and warmed at 37°C for 5 minutes before the addition of 0.5 mL of labelled PMN suspensions containing of 2 × 10^6^ PMN. The cells were then incubated at 37°C for 30 minutes. Thereafter, the cells were washed 4 times with PBS, and 250 *μ*L of 1% Triton X-100 in PBS were then added. Culture dishes were shaken for 15 minutes at room temperature and supernatant was removed. Fluorescence was measured immediately at 520 nm excitation-light and 565 nm emission-light. The results were recorded as a number of PMN adhered, calculated from a standard curve generated by measuring the fluorescence of different known number of labelled PMN processed identically as the adhesion samples. 

For the transendothelial migration assays, tumour cells, dermal fibroblasts and HMVEC were cultured in the 12-well tissue culture plates. When the cells were confluent, they were washed twice with PBS and then incubated with 1 mL of DMEM, free of pH indicator (to avoid colour interference in the fluorescence measurement) containing 10% FBS, for 48 hours in a CO_2_ incubator. HMVEC were treated with IL-1*β* for the final 5 hours. Transendothelial migration assays were performed placing the 3 *μ*m pore size cell culture inserts (BD Labware Europe, Le Pont de Claix, France) in which HMVEC were previously cultured at confluence. The assay was performed without removing conditioning media from the wells containing tumour cells, fibroblasts and IL-1*β*-treated HMVEC. The medium from the inserts was removed and 2 × 10^6^ PMN in 500 *μ*L of 37°C pre-warmed DMEM free of pH indicator containing 10% FBS were then added. The system was placed in the CO_2_ incubator and PMN were allowed to cross the HMVEC monolayer for 3 hours. After this, inserts were removed and 100 *μ*L of 1% Triton X-100 in PBS were added to the bottom wells. Samples were processed as described above to determine the number of PMN that crossed. After washing integrity of HMVEC layer was routinely controlled by microscopy observation and no alterations were observed in any case. Non-specific transmigration was evaluated without cells in the bottom wells and subtracted from the values obtained with cells.

### 2.5. Cytotoxicity Assay

Tumour cells were cultured in 12-well culture plates containing 12 mm diameter cover glasses. Before the assay, tumour cells were washed and 500 *μ*L of DMEM containing 10% FBS were added. 5 × 10^6^ PMN suspended in 500 *μ*L of DMEM with 10% FBS were placed in 0.4 *μ*m and 3 *μ*m pore cell culture inserts that were located over the wells containing the tumour cells. Four different conditions were assayed: PMN placed in 3 *μ*m inserts; PMN activated with 0.1 *μ*moles/L n-formyl-methionyl-leucyl-phenylalanine (fMLP) placed in 0.4 *μ*m inserts; PMN placed in 3 *μ*m inserts in the presence of a cocktail of 10 *μ*g/mL of mouse anti-human CD44 monoclonal antibody (ref 550990, BD Pharmingen, San Diego, CA), 10 *μ*g/mL of mouse anti-human CD162 monoclonal antibody (ref 556052, BD Pharmingen) and 10 *μ*g/mL of mouse anti-human CD18 monoclonal antibody (ref 555922, BD Pharmingen) and controls were performed with medium without PMN in the inserts. PMN were incubated for 30 minutes with the antibody cocktail before placing them in the inserts. Plates containing all of the cells were incubated for the indicated (results section) period of time in the CO_2_ incubator. Covers were recovered for microscopic observation after acridine orange (AO) and ethidium bromide (EB) staining. For staining, covers were treated with a 1 *μ*g/mL AO and 5 *μ*g/mL EB solution in PBS for 15 minutes and mounted with Fluoprep (BioMérieux, France) in glass slides for microscopy, and observed in an Olympus BX50 microscope. AO permeates throughout the cells and renders the nuclei green. EB was taken up by the cells only when cytoplasmic membrane integrity was lost, and stained the nuclei red.

### 2.6. Protein Array Test

Secreted cytokines were characterized with the Human Cytokines Antibody Array 3 (RayBiotech, Inc., Norcross, GA30092, 1-888-494-8555) and samples were processed as the protocol provided by the manufacturer. Detection was afforded by incubating 0.5 mL of mixture of the ECL Western Blotting Analysis System (GE Healthcare, Buckinghamshire HP79NA UK) for 1 minute. X-ray film used was Curix RP2 and were developed with G153 developer and Rapid Fixer G354 (Agfa-Gevaert, B2640 Mortsel, Belgium). The density of blots was measured in a GelDoc 2000 with the Quantity One software (Bio-Rad Laboratories, Hercules, CA).

### 2.7. Quantitative Protein Analysis

Quantitative analysis of the selected proteins in the culture media were performed by specific ELISA following the manufacturer's instructions (GRO*α* and ENA-78 were from R&D Systems, Minneapolis, MN; IL-8 was from Endogen-Pierce, Rockford, IL; VEGF was from Biosource Europe, Fleurus, Begium).

### 2.8. Flow Cytometry Analysis of Surface ICAM-1 Expression on HMVEC

HT-29 and FaDu were cultured at confluence as aforementioned. The medium was then replaced, and after 48 hours, it was recovered and conditioning medium stored at −80°C until used for HMVEC stimulation. Stimulation of confluent HMVEC was performed by replacing the medium by DMEM containing 10% FBS (control), conditioning medium from tumour cells or DMEM containing 10% FBS and 10 U/mL human recombinant IL-1*β* (positive control, Roche Applied Science, Barcelona, Spain). After incubation overnight, HMVEC were detached with Cell Dissociation Solution (Sigma, St Louis, MO) centrifuged and suspended in PBS pH 7.4 containing 2% of bovine serum albumin and 0.1% sodium azide. Twenty *μ*L of commercial solution of R-phycoerythrin (PE) labelled mouse anti-human ICAM-1 (CD54, ref 555511, BD Pharmingen) were then added and allowed to stand at 4°C in darkness for 20 minutes. Cells were then centrifuged, washed twice and suspended in 400 *μ*L PBS containing 2% paraformaldehide. Flow cytometry analysis was performed in a Cytomics FC500 cytometer (Beckman-Coulter, Miami, FL).

## 3. Results

To observe the effect of PMN on tumour cells, the tumour cell lines were cultured in the bottom wells of transwell devices; 5 × 10^6^ PMN were added via the upper membrane pores. The system was then left in a culture chamber overnight. To determine the dependence of the cytotoxic effect of PMN on contact between the latter and tumour cells, two kind of insert were used. In one set of experiments PMN were placed on 3 *μ*m pore inserts which allow PMN to pass through. These experiments were performed in the absence and presence of a cocktail of antibodies against CD18, CD162 and CD44 to prevent the firm adhesion of the PMN to the tumour cells. In a second set of experiments, PMN were placed on 0.4 *μ*m pore inserts to prevent their passage; these membranes, however, allow mediator molecules to pass through. In these experiments, the PMN were exogenously stimulated by adding fMLP to the inserts. Control experiments consisted of placing culture medium without PMN on the inserts.

**Figure 1:**
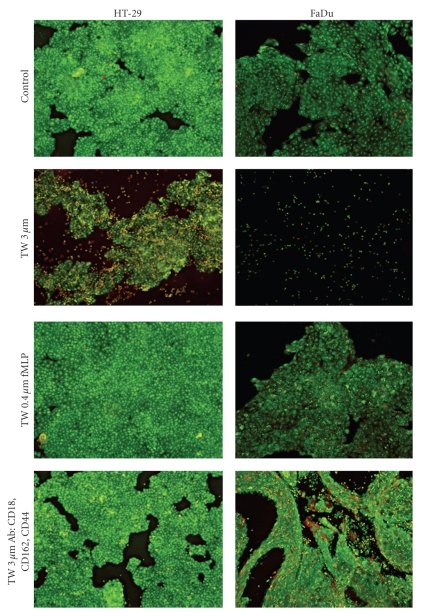
Representative microscopic photographs (×100) of tumour cells exposed overnight to PMN. Tumour cells were cultured in 12-well culture plates containing 12 mm diameter cover glasses. 5 × 10^6^ PMN were placed on culture inserts that were located over the wells containing the tumour cells. Control, medium without PMN in the inserts; TW 3 *μ*m, PMN placed in 3 *μ*m pore size inserts; TW 0.4 *μ*m fMLP, PMN activated with 0.1 *μ*moles/L fMLP placed in 0.4 *μ*m pore size inserts; and TW 3 *μ*m Ab:CD18, CD162, CD44, PMN placed in 3 *μ*m pore size inserts with 10 *μ*g/mL of mouse anti-human CD44 monoclonal antibody, 10 *μ*g/mL of mouse anti-human CD162 monoclonal antibody and 10 *μ*g/mL of mouse anti-human CD18 monoclonal antibody. Covers were then stained with AO-EB. AO permeates throughout the cells and renders the nuclei green. EB is taken up by the cells only when cytoplasmic membrane integrity is lost, and stains the nuclei red. *N* = 3 with similar results.

**Figure 2:**
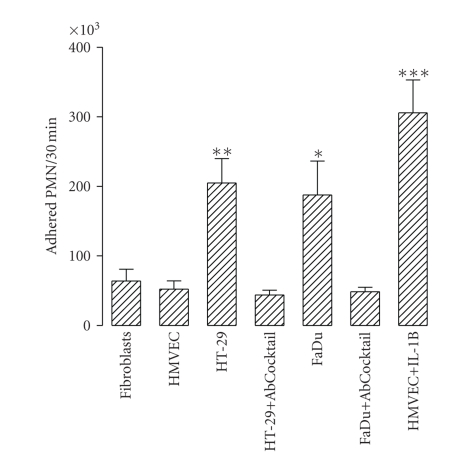
Adhesion of PMN to tumour cells. 2 × 10^6^ DiI-labeled PMN were added to culture wells containing confluent dermal fibroblasts (fibroblasts), HMVEC (HMVEC), HT-29 (HT-29), HT-29 with 10 *μ*g/mL of mouse anti-human CD44 monoclonal antibody, 10 *μ*g/mL of mouse anti-human CD162 monoclonal antibody and 10 *μ*g/mL of mouse anti-human CD18 monoclonal antibody in the medium (HT-29 + AbCocktail), FaDu (FaDu), FaDu with the Ab Cocktail (FaDu + AbCocktail), and HMVEC previously treated with 10 U/mL of human recombinant IL-1*β* for 5 hours (HMVEC + IL-1B) and incubated at 37°C for 30 minutes. After washing, adhered PMN were evaluated as described in the Methods Section. Bars represent the mean ± SEM of 7 independent experiments performed by sixtoplicate. Statistical significance was assessed using ANOVA test; *P < .05, **P < .01, and ***P < .001 when compared with HMVEC group.

As expected, a cytotoxic effect of PMN on the two cultured cell lines was observed when PMN passed freely through the upper inserts. A representative experiment is shown in Figure 1. FaDu cells were more sensitive to PMN than were HT-29 cells, whereas the complete destruction of the cultured FaDu colonies was observed after overnight incubation with PMN, 48 hours of incubation was necessary for the complete destruction of HT-29 cultures (not shown). When PMN were unable to pass through the membrane (inserts pore diameter 0.4 *μ*m) no destruction of HT-29 cell aggregates was observed even when PMN were stimulated with fMLP; in addition, the cytotoxic effect on FaDu was clearly mitigated. Even when PMN crossed the membrane (3 *μ*m diameter pore) a similarly reduced cytotoxic effect was observed when these PMN were in a cocktail of antibodies directed against adhesion molecules (thus inhibiting the adhesion of PMN to the tumour cells) (Figure 2). These results indicate that direct contact and adhesion between PMN and tumour cells is important, although not absolutely necessary in the case of FaDu, for these immune system cells to exert their cytotoxic effect.

To examine the ability of tumour cells to adhere to PMN, 2 × 10^6^ DiI-labelled PMN were directly added to culture wells containing confluent dermal fibroblasts or HMVEC (negative controls), tumour cells, tumour cells with the cocktail of antibodies against adhesion molecules, and HMVEC previously treated with 10 U/mL of human recombinant IL-1*β* for 5 hours (positive control); all were incubated at 37°C for 30 minutes. The results in Figure 2 show that PMN adhered to both HT-29 and FaDu cells. The adhesion of PMN to tumour cells was significantly greater than their adhesion to dermal fibroblasts and unstimulated HMVEC. Treatment of PMN with the antibody-cocktail against PMN adhesion molecules strongly reduced their adhesion to tumour cells.

**Figure 3:**
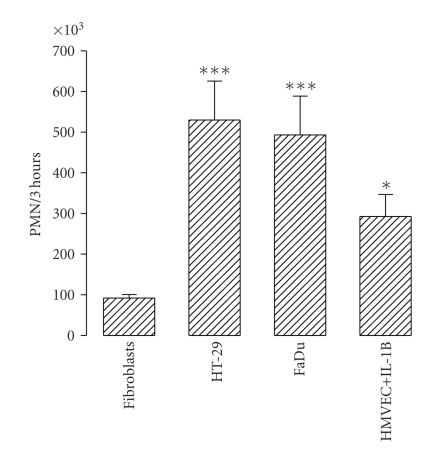
Tumour cell-induced PMN transmigration through human microvascular endothelium. DiI-labeled PMN were added to 3 *μ*m pore size cell culture inserts previously coated with HMVEC cultured at confluence. Inserts were placed upon culture wells containing confluent human dermal fibroblast (fibroblasts), HT-29 (HT-29), FaDu (FaDu) and HMVEC previously treated with 10 U/mL of human recombinant IL-1*β* for 5 hours (HMVEC + IL-1B). See Methods. After 3 hours at 37°C, PMN in the lower side of the system were evaluated as described in the Methods section. Bars represent the mean±SEM of 7 independent experiments performed by sixtoplicate. Statistical significance was assessed using ANOVA test; *P < .05, and ***P < .001 when compared with fibroblasts group.

The ability of tumour cells to induce PMN transmigration through a human microvascular monolayer was examined by adding DiI-labelled PMN to 3 *μ*m pore size cell culture inserts previously coated with HMVEC cultured until confluence. Inserts were placed on culture wells containing confluent human dermal fibroblast (negative control), tumour cell lines, and HMVEC previously treated with 10 U/mL of human recombinant IL-1*β* for 5 hours (positive control). The transwell systems were then incubated at 37°C for 3 hours. Figure 3 shows the number of PMN that migrated toward the tumour cells in the bottom wells over the 3 hours period. The number of PMN that crossed the HMVEC layer was significantly higher when the tumour cells were in the bottom than when dermal fibroblasts were in this location. Tumour cells attracted PMN more so than did HMVEC stimulated with IL-1*β*.

**Figure 4:**
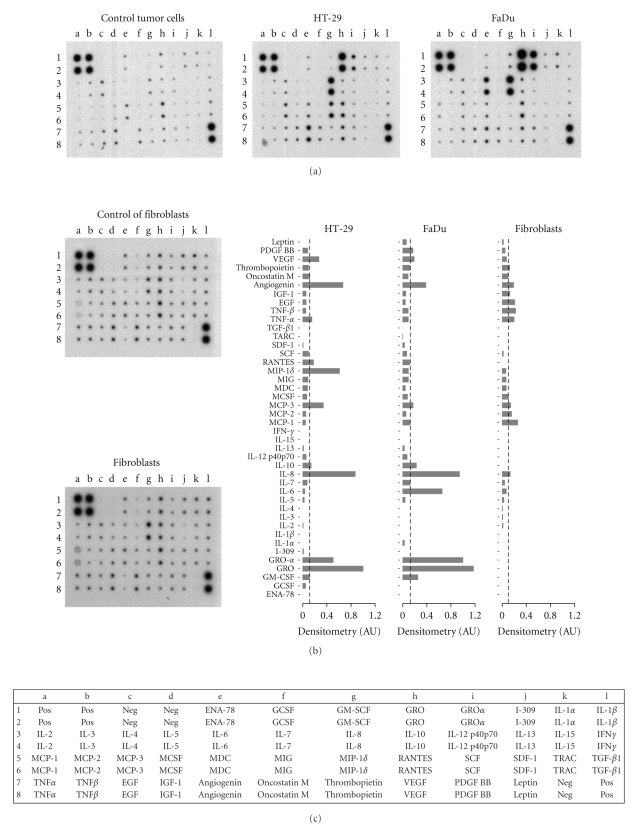
Protein array analysis of culture medium from HT-29, FaDu and human dermal fibroblasts. Culture medium (DMEM plus 10% FBS) was replaced and collected after 48 hours. Controls were performed with DMEM plus 10% FBS. The proteins that were analyzed are indicated in the bottom panel; pos, positive controls, neg, negative controls. Bars are the densitometric evaluation of samples (right panel); AU, arbitrary units. Values were calculated as the difference between the density value of samples and controls. Bars represent the mean of two independent experiments. Doted lines indicate the value which comprise 75% of the proteins.

**Figure 5:**
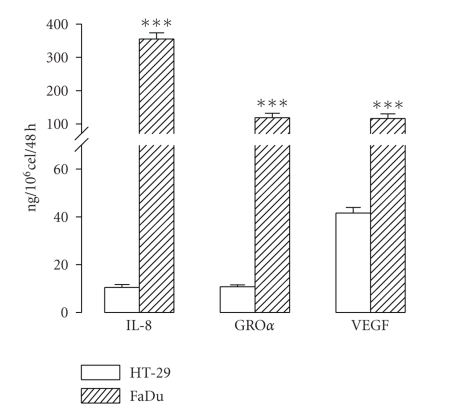
Quantitative evaluation of the IL-8, growth related oncogene-*α* (GRO*α*), and vascular endothelial growth factor (VEGF) released by HT-29 and FaDu in 48 hours. Culture medium (DMEM plus 10% FBS) was replaced and collected after 48 hours. Protein levels were determined by ELISA. Bars represent the mean ± SEM, *n* = 4. Statistical significance was assessed using Students t-test;***P < .001 when compared with HT-29.

A nonquantitative protein expression array was used to explore the cytokines and growth factors released by the tumour cell lines. Human dermal fibroblasts were included in the analysis since these cells were used as negative controls in many experiments. The results are shown in Figure 4. Conditioning media from tumour cell cultures were positive for many proteins. Densitometry evaluation of the array spots was performed to estimate the relative expression of the proteins evaluated (also shown in Figure 4). The major chemokines released by both tumour cell lines were the CXC-chemokines GRO*α* and IL-8. Quantitative evaluation of GRO*α*, IL-8 and VEGF by ELISA showed that both HT-29 and FaDu cells released substantial amounts of these proteins, although levels in the FaDu conditioning medium were an order of magnitude greater (Figure 5). 

**Figure 6:**
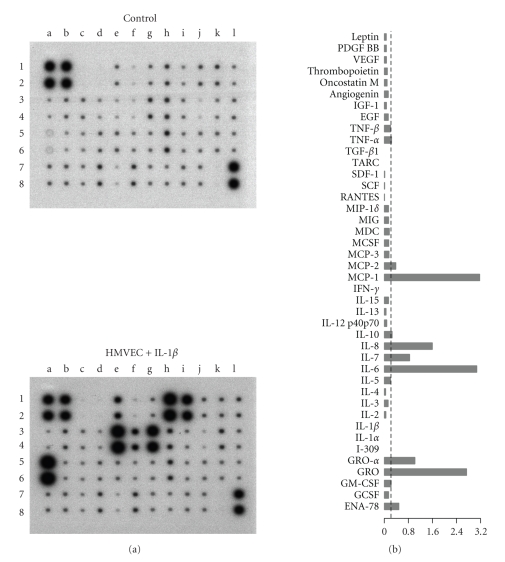
Protein array analysis of culture medium from HMVEC exposed to IL-1*β*. Culture medium was replaced by DMEM plus 10% FBS and 10 U/mL of human recombinant IL-1*β*, and collected after 48 hours. Controls were performed with DMEM plus 10% FBS and 10 U/mL of human recombinant IL-1*β*. The proteins that were analyzed are indicated in the bottom panel of Figure 4. Densitometric evaluation of samples is also shown (right panel). Values were calculated as the difference between the density value of samples and controls. Columns represent the mean of 2 independent experiments. The density value which comprise 75% of the proteins is indicated by the doted line. AU, arbitrary units.

**Figure 7:**
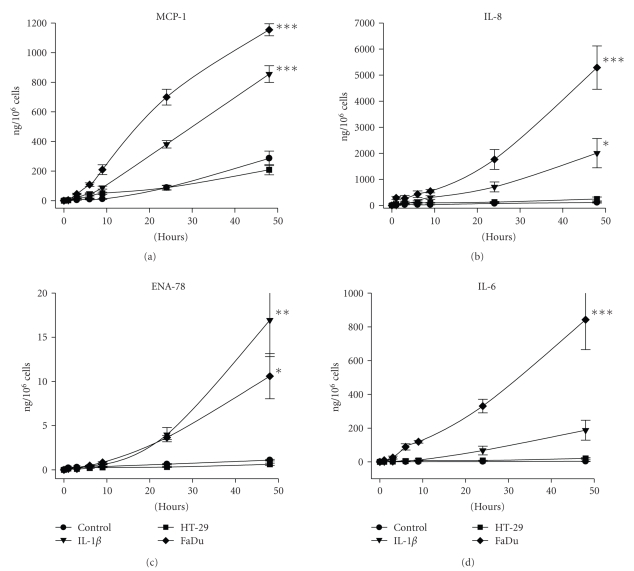
Production of cytokines by HMVEC stimulated with tumour cell conditioning medium. HMVEC cultures were exposed to 48 hours-conditioning medium of HT-29 and FaDu for the indicated periods of time. Production of cytokines by HMVEC was calculated as the difference between the value of samples and the initial amount in the conditioning mediums from tumour cells. Bars represent the mean ± SEM of 5 independent experiments performed by triplicate. Statistical significance was assessed using multivariate Wilks statistic and one sided Dunnet test to compare every treatment groups with the control group; *P < .05, **P < .01 and ***P < .001.

To determine the ability of tumour cells to activate microvascular endothelium, HMVEC were subjected to conditioning media from the tumour cell line cultures. First, the profile of cytokines and growth factors released by HMVEC was determined using a protein array assay (Figure 6). Culture medium from HMVEC stimulated with IL-1*β* was clearly positive for MCP-1, IL-8, GRO*α* and IL-6. In addition, ENA-78, which was not released by the tumour cells (see Figure 4), was specifically released by HMVEC. The time course of the release of MCP-1, IL-8, ENA-78 and IL-6 by HMVEC exposed to IL-1*β* and conditioning media from tumour cell lines is shown in Figure 7. IL-8 was the major cytokine produced by the HMVEC. Conditioning medium from HT-29 did not stimulate HMVEC to release any protein for which tests were made. In contrast, conditioning medium from FaDu cultures strongly induced HMVEC to release the four cytokines. The production of MCP-1, IL-8 and IL-6 induced by the FaDu conditioning medium was even stronger than that induced by IL-1*β*. 

**Figure 8:**
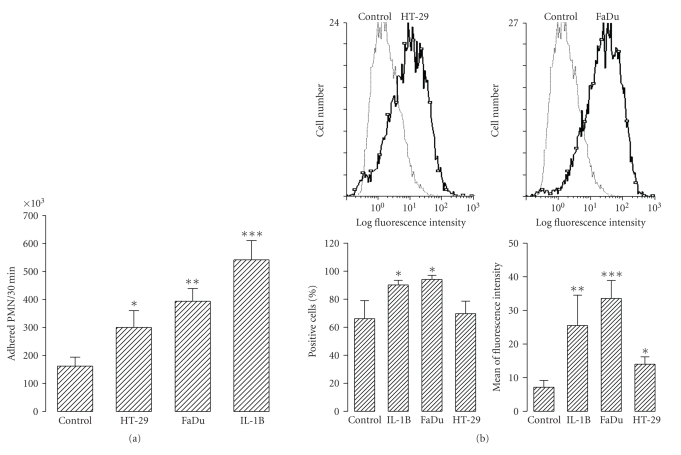
(a) Adhesion of PMN to HMVEC exposed to tumour cell conditioning medium. 2 × 10^6^ DiI-labeled PMN were added to the culture wells containing confluent HMVEC previously treated overnight with none (control), HT-29 (HT-29) and FaDu (FaDu) 48 hours conditioning medium, and 10 U/mL of human recombinant IL-1*β* (IL-1B) and then incubated at 37°C for 30 minutes. After washing, adhered PMN were evaluated as described in the Methods section. Bars represent the mean±SEM of 7 independent experiments performed in sixtoplicate. (b) Surface expression of ICAM-1 in HMVEC exposed to tumour cell conditioning media. HMVEC previously treated overnight with none (control), HT-29 (HT-29) and FaDu (FaDu) 48 hours conditioning medium, and 10 U/mL of human recombinant IL-1*β* (IL-1B) were detached and analyzed for ICAM-1 expression by flow cytometry (see Methods). Upper panels represent histograms (out of 4) showing the effect of tumour cell conditioning medium on ICAM-1 expression. Bottom panels show quantitative data. Bars represent the mean ± SEM of 4 independent experiments. Statistical significance was assessed using ANOVA test; *P < .05, **P < .01, and ***P < .001 when compared to control group.

To observe the effect of tumour cell conditioning media on the capacity of HMVEC to adhere to PMN, HMVEC were exposed overnight to fresh culture medium (negative control), conditioning media from tumour cells, or 10 U/mL of human recombinant IL-1*β* (positive control) before the adhesion test was performed. After washing, 2 × 10^6^ DiI-labelled PMN were added to the HMVEC and then incubated at 37°C for 30 minutes. Conditioning medium from tumour cells significantly increased the adhesion of PMN to HMVEC (Figure 8(a)). Once the value of the adhesion of PMN to control HMVEC was subtracted, the FaDu conditioning medium showed an almost 2-fold capacity to increase the ability of HMVEC to adhere to PMN than did the HT-29 conditioning medium. This was in agreement with the induction of ICAM-1 (Figure 8(b)). The percentage of positive HMVEC cells (Figure 8(b), bottom left panel) treated with FaDu conditioning medium or IL-1 (positive control) was similar, whereas the percentage of positive HMVEC treated with HT-29 conditioning medium was similar to that obtained with the untreated controls. However, differences in the percentage of positive HMVEC cells treated with FaDu conditioning medium or IL-1*β* and the controls were significant (although not very important). This means that many untreated HMVEC express ICAM-1 constitutively (about 70% of the control cells and HT-29-treated HMVEC were positive) and that the number of cells expressing ICAM-1 was not increased much by treatment with FaDu conditioning medium or IL-1*β* (about 90% FaDu- and IL-1-treated HMVEC were positive). Nevertheless, with respect to the mean fluorescence intensity, which is related to the density of ICAM-1 (number ICAM-1 molecules/positive cell), the treatment of HMVEC with FaDu or HT-29 conditioning medium significantly increased ICAM-1 expression in these cells (although the FaDu conditioning medium was much more potent in this respect; Figure 8(b), right bottom panel).

## 4. Discussion

The major aim of our study was to test the hypothesis that tumour cells have proinflammatory properties and produce cytokines and growth factors that condition the tumours for attack by PMN. Our results are consistent with many reports showing PMN cytotoxicity against tumour cells [3–9]. We observed that the tumour cell lines that we used were sensitive to PMN. When direct contact or adhesion of PMN to tumour cells did not occur, cell lysis was drastically reduced indicating that adhesion was an important condition for the cytotoxic effect of PMN on tumour cells. This is consistent with data reported by others [27, 36]. 

We hypothesized that PMN action against tumours in vivo requires that the tumour cells have the ability to act as an inflammatory site. Obviously, the first condition that tumour cells must fulfil is to be able to attract PMN. Both of the tumour cell lines (HT-29 and FaDu) that we used accomplished this by secreting considerable amounts of CXC chemokines such as IL-8, and GRO*α*. These results are consistent with the finding that many tumour cell lines constitutively produce IL-8 and other chemokines [47, 48]. It is noteworthy that the major chemokines produced by these HT-29 and FaDu (IL-8 and GRO*α*) act mainly on PMN [49–51]. Constitutive production of chemokines by tumour cells may be related to the constitutive activation of NF-*κ* *B* found on many tumour types [52, 53]. The production of IL-8 by tumour cells has been interpreted usually as tumorigenic due to its pro-angiogenic and pro-metastatic properties [54–57]. Nevertheless, the major biological activity of IL-8 is to attract and activate leukocytes, particularly PMN [58–60] to the inflammation site and this activity could be potentially antitumoral. The results from our transendothelial migration experiments indicated that the concentration of PMN attractants in the conditioning media of both HT-29 and FaDu was sufficient to recruit PMN effectively. 

In vivo recruitment of leukocytes is mediated by activated vascular endothelium [61]. So, tumour cells should be able to activate EC. We have shown that HT-29 and FaDu functionally induced adhesion of PMN to HMVEC. This correlated with induction by the tumour cells of ICAM-1 expression on the surface of HMVEC, which is an essential adhesion molecule for surface attachment and transcellular migration of PMN through vascular endothelium [62]. ICAM-1 is the counter receptor for PMN *β*_2_-integrins [63]. These integrins on the PMN surface are essential for adhesion and PMN-mediated cytotoxicity [64, 65]. Regarding activation of HMVEC to secrete chemokines, we observed a great difference between HT-29 and FaDu. The former did not induced significant production of cytokines by the HMVEC, whereas the later dramatically induced production of chemokines and IL-6 by HMVEC. Moreover, the expression of MCP-1, IL-8 and IL-6 was much higher in response to FaDu conditioning medium than after treatment of HMVEC with IL-1*β*. These results strongly suggest that there are differences between different tumours regarding their ability to promote an inflammatory response. The nature of these differences might explain the efficacy of some tumours to act as sites of inflammation. Also, such knowledge would help to design a PMN-based anticancer therapy.

It is widely accepted that the elimination of any cancer is possible by activating the host immune response [66]—but this has not yet been translated into clinical practice. Despite the increasing evidence demonstrating that innate immunity, mainly associated with PMN, has an impressive antitumoural capacity [26, 39–41, 67, 68], current immuno-therapeutic developments in the field of cancer are largely based upon the stimulation of adaptive immunity [66, 69]. Adaptive mechanisms usually play a primary role in initiating tissue-specific reactions that recruit innate effector cells, such as PMN, to the target organ, but these latter cells are primarily responsible for tumour rejection [70]. The efferent arm of the immune system, mainly PMN, requires activation by appropriate signals at the tumour site to undertake its functions. Established cancers usually either do not send out such signals or send out signals insufficiently potent to initiate and sustain a powerful inflammatory response, and thus induce their own rejection. Evidence is growing that the elimination of tumours using immunological “weapons” is only possible by significantly enhancing an acute inflammatory response. There is probably a threshold number of PMN required for their antitumour activity to be effective, and a simple way of achieving this is to increase their circulating levels, as described by Brú et al. [37–40]. 

The present work was performed with only two established tumour cell lines, but the results open up interesting possibilities. If malignant tumours share the ability to activate the natural mechanisms of local PMN recruitment, it might be possible to produce an acute local inflammatory response at tumour sites. PMN-based anticancer therapy may be appropriate for tumours that that do not induce a sufficiently strong immune response on their own. Very recently, we reviewed the literature showing evidence that many solid tumours can be destroyed by PMN in vitro and/or in vivo [71].
